# Efficacy and safety outcomes of patients with atrial fibrillation compared between warfarin and non-vitamin K antagonist oral anticoagulants based on SAMe-TT_2_R_2_ score

**DOI:** 10.1186/s12872-023-03053-w

**Published:** 2023-01-23

**Authors:** Komsing Methavigul, Ahthit Yindeengam, Rungroj Krittayaphong

**Affiliations:** 1 Department of Cardiology, Central Chest Institute of Thailand, Nonthaburi, Thailand; 2 Her Majesty Cardiac Center, Faculty of Medicine Siriraj Hospital, Mahidol University, Bangkok, Thailand; 3 Division of Cardiology, Department of Medicine, Faculty of Medicine Siriraj Hospital, Mahidol University, 2 Wanglang Road, Bangkoknoi, Bangkok, 10700 Thailand

**Keywords:** Warfarin, NOACs, Low SAMe-TT_2_R_2_, Atrial fibrillation

## Abstract

**Objectives:**

This study aimed to investigate the efficacy and safety outcomes of patients with atrial fibrillation (AF) compared between those taking warfarin and non-vitamin K antagonist oral anticoagulants (NOACs) based on SAMe-TT_2_R_2_ score.

**Methods:**

AF patients using warfarin or NOACs were enrolled from Thailand’s COOL-AF registry. A low SAMe-TT_2_R_2_ score was defined as a score of 0–2. The efficacy outcomes were all-cause death, ischemic stroke (IS), transient ischemic attack (TIA), and/or systemic embolization (SE). The safety outcome was major bleeding (MB). The secondary outcome was a combination of cardiovascular (CV) death, IS/TIA/SE, or MB. Cox proportional hazards model was used to compare the event rate between the AF patients taking warfarin and NOACs according to SAMe-TT_2_R_2_ score.

**Results:**

A total of 2568 AF patients taking oral anticoagulants were enrolled. Warfarin and NOACs were used in 2340 (91.1%) and 228 (8.9%) patients, respectively. Among overall patients, 305 patients taking warfarin (13.0%) and 21 patients taking NOACs (9.2%) had the efficacy outcome, while 155 patients taking warfarin (6.6%) and 11 patients taking NOACs (4.8%) had the safety outcome. After adjustment for confounders, overall patients taking warfarin had significantly more secondary outcome than those taking NOACs (11.4% vs. 7.5%, respectively; adjusted hazard ratio: 1.74, 95% confidence interval: 1.01–2.99; *p* = 0.045) regardless of SAMe-TT_2_R_2_ score.

**Conclusions:**

AF patients taking warfarin had a significantly higher CV death or IS/TIA/SE or MB compared to those taking NOACs regardless of SAMe-TT_2_R_2_ score. The results of this study do not support the use of SAMe-TT_2_R_2_ score to guide OAC selection.

## Introduction

Acute ischemic stroke is the most catastrophic complication in patients with non-valvular atrial fibrillation (AF). Oral anticoagulants (OACs) are recommended in AF patients with a CHA_2_DS_2_-VASc score of 1 or more in males, and 2 or more in females, respectively [1–3].

There are currently two groups of OACs—vitamin K antagonists (VKAs), such as warfarin, and non-vitamin K antagonist oral anticoagulants (NOACs). Warfarin is the most common VKA used in Thailand, but it has some limitations in clinical practice due to necessitate of international normalized ratio (INR) monitoring. The therapeutic range of INR ranges from 2 to 3 [4–6] despite the findings of a previous study conducted in Thailand that recommended a lower therapeutic INR range [7]. Data from the COhort of antithrombotic use and Optimal INR Level in patients with non-valvular AF in Thailand (COOL-AF Thailand) registry strongly suggest that a lower therapeutic INR is needed in older adult patients [8]. Moreover, results from a study that was conducted in Thailand showed the optimal INR in AF patients with evaluated heart valves, rheumatic or artificial (EHRA) type 2 valvular heart disease to be 2.00–2.49 [9].

Rhythm control of AF by catheter ablation had a high success rate and might open the opportunity for the discontinuation of OAC to avoid the adverse effect of OAC [10]. Early rhythm control by radiofrequency ablation compared to drug treatment can reduce the risk of clinical composite outcome [11]. Recent advances in the development of ablation strategy such as cryoablation have shown that cryoablation can be the initial treatment option of patients with AF [12]. Meta-analyses of radiofrequency ablation compared to anti-arrhythmic drug indicated that radiofrequency ablation is superior to drug treatment [13]. Similar result has been reported from meta-analysis of patients with AF with heart failure [14]. However, a recent guideline suggested OAC for at least 2 months after AF ablation and long-term OAC is recommended according to the CHA_2_DS_2_-VASc score [1].

Poor time in therapeutic range (TTR) is another problem that is common among AF patients taking warfarin. Use of the SAMe-TT_2_R_2_ score was recently proposed to predict poor TTR [15–22]. Recent European guidelines recommend considering VKA or NOACs if a patient’s SAMe-TT_2_R_2_ score is within the range of 0–2 [1]. The aim of this study was to investigate the efficacy and safety outcomes of patients with atrial fibrillation (AF) compared between those taking warfarin and those taking NOACs based on SAMe-TT_2_R_2_ score.

## Methods

Patients with AF were prospectively recruited from 27 hospitals in Thailand during 2014–2020 into the COhort of antithrombotic use and Optimal INR Level in patients with non-valvular Atrial Fibrillation in Thailand (COOL-AF Thailand) registry. The selection of 27 hospitals was based on the geographic distribution of the hospitals to cover all regions of Thaialnd and also based on the university-based and government-based hospitals which had difference in hospital size and practices. The enrollment period of this study was 2014–2017.

COOL-AF registry is a multicenter, prospective cohort of patients with non-valvular atrial fibrillation. Primary objective of the registry is to determine antithrombotic pattern, and to identify optimal INR for Thai population, and clinical outcomes. The original description of the study protocol was previously published [23]. Patients aged 18 years or more were enrolled in this prospective cohort study. AF was diagnosed by standard electrocardiography (ECG) or ambulatory monitoring. Patients with prosthetic heart valve, rheumatic mitral valve disease, recent ischemic stroke within 3 months, transient reversible cause of AF, life expectancy less than 3 years, pregnancy, thrombocytopenia, myeloproliferative diseases were excluded from this study. Protocols were established and followed by the data management team and statisticians to ensure the integrity and quality of the data before final analysis.

The protocol for this study was approved by the Central Research Ethics Committee (CREC) and the Institutional Review Boards of each participating hospital. Written informed consent was obtained from all study patients. This study was in compliance with the International Conference on Harmonization for Good Clinical Practice Guidelines (ICH-GCP), and with the principles set forth in the 1964 Declaration of Helsinki and all of its subsequent amendments.

### Data collection

All investigators were instructed to enroll patients consecutively to minimize the selection bias. The following data were collected after the informed consent process: demographic, weight, height, vital signs, AF duration and symptom, medical history, concomitant diseases such as diabetes, hypertension, physical examination, medications, laboratory data, ECG and investigational lab data, and components of CHA_2_DS_2_-VASc and HAS-BLED score. The SAMe-TT_2_R_2_ score was classified as low score (score range: 0–2) or high (score range: 3–8). Patient data were recorded at follow-up visits scheduled for every 6 months. For follow-up visits, the data were recorded similar to the baseline visit. Clinical outcome data were recorded during the follow-up visit. According to the study protocol, site investigators were instructed to record follow-up data at 6, 12, 18, 24, and 30 months with an allowable window of ± 1 month.

Each component of the SAMe-TT_2_R_2_ score was scored and recorded as S = female sex (1 point); A = age < 60 years (1 point); Me = medical history > 2 of the following: hypertension, diabetes, coronary artery disease (CAD)/myocardial infarction (MI), peripheral arterial disease, congestive heart failure, previous stroke, pulmonary disease, and hepatic or renal disease (1 point); T = treatment (interacting drugs, e.g., amiodarone for rhythm control) (1 point); T_2_ = tobacco use within 2 years (2 points); and, R_2_ = non-Caucasian race (2 points).

### Clinical outcomes

The primary efficacy outcome was all-cause death, ischemic stroke (IS)/transient ischemic attack (TIA), and/or systemic embolization (SE). IS was defined as a sudden onset of neurological deficit that lasted at least 24 h, but with no evidence of intracranial hemorrhage (ICH) by computed tomography (CT) or magnetic resonance imaging (MRI) of the brain [24]. TIA was defined as a sudden onset of neurological deficit that lasted less than 24 h [24]. SE was defined as disruption of blood flow to other arteries, such as acute limb arterial occlusion or acute mesenteric arterial occlusion [25].

The primary safety outcome was major bleeding, including extracranial major bleeding and/or ICH. Major bleeding was defined as fatal bleeding; critical organ bleeding, including ICH, intraspinal, intraocular/retinal, retroperitoneal, intraarticular, pericardial, intramuscular with/without compartment syndrome; and/or, bleeding that caused a decrease in hemoglobin level of 2 g/dL or more, or that resulting in a need for blood transfusion of 2 or more units of blood.

The secondary outcomes were cardiovascular (CV) death, the combination of CV death or IS/TIA and/or SE, and the combination of CV death, IS/TIA/SE, or major bleeding. A CV death was defined as IS/TIA, MI and/or SE.

### Statistical analysis

Descriptive statistics were used to summarize patient demographic and clinical characteristics in this study. Categorical data were compared using chi-square test, and those results are given as frequency and percentage. Continuous data (all of which were normally distributed) were compared using Student’s t-test, and those results are shown as mean ± standard deviation (SD). Cox proportional hazards model was used to compare the event rate of primary efficacy, primary safety, and the secondary outcome between the AF patients taking warfarin and the patients taking NOACs according to SAMe-TT_2_R_2_ score (low or high). The results of those analyses are presented as hazard ratio (HR) and 95% confidence interval (CI). The baseline variables that were used for adjustment in the models included age, sex, diabetes, hypertension, history of CAD/previous myocardial infarction, history of heart failure, history of ischemic stroke/TIA, serum creatinine, left ventricular ejection fraction (LVEF). During the multivariable analysis, backward elimination with *p* value < 0.05 as the stopping criteria was used. Cox proportional hazards model results after adjustment for potential confounders are shown as adjusted HR and 95%CI. All statistical analyses were performed using SPSS Statistics software (SPSS, Inc., Chicago, IL, USA), and a *p* value less than 0.05 was considered statistically significant for all tests.

## Results

**Fig. 1:**
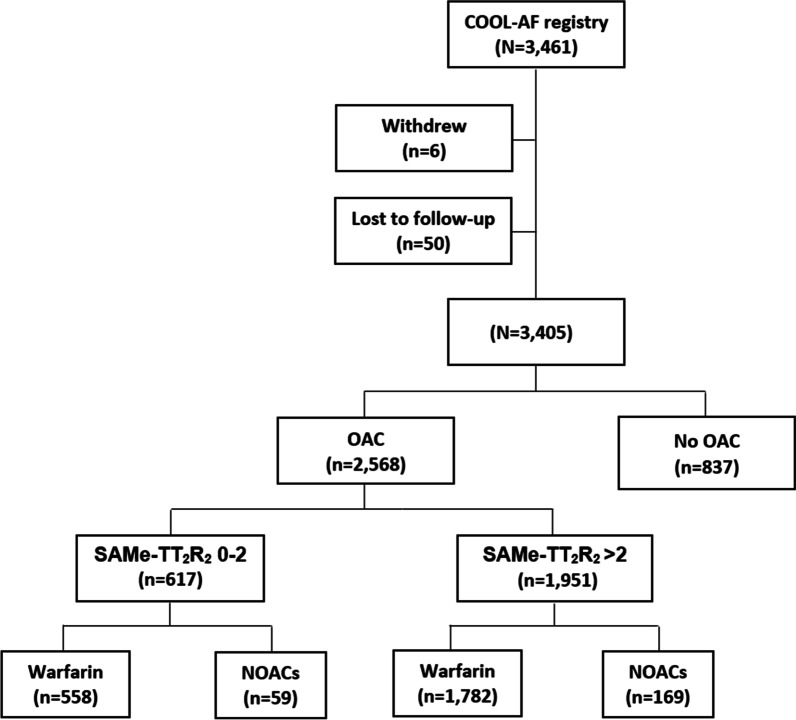
Flow diagram of study population

**Fig. 2:**
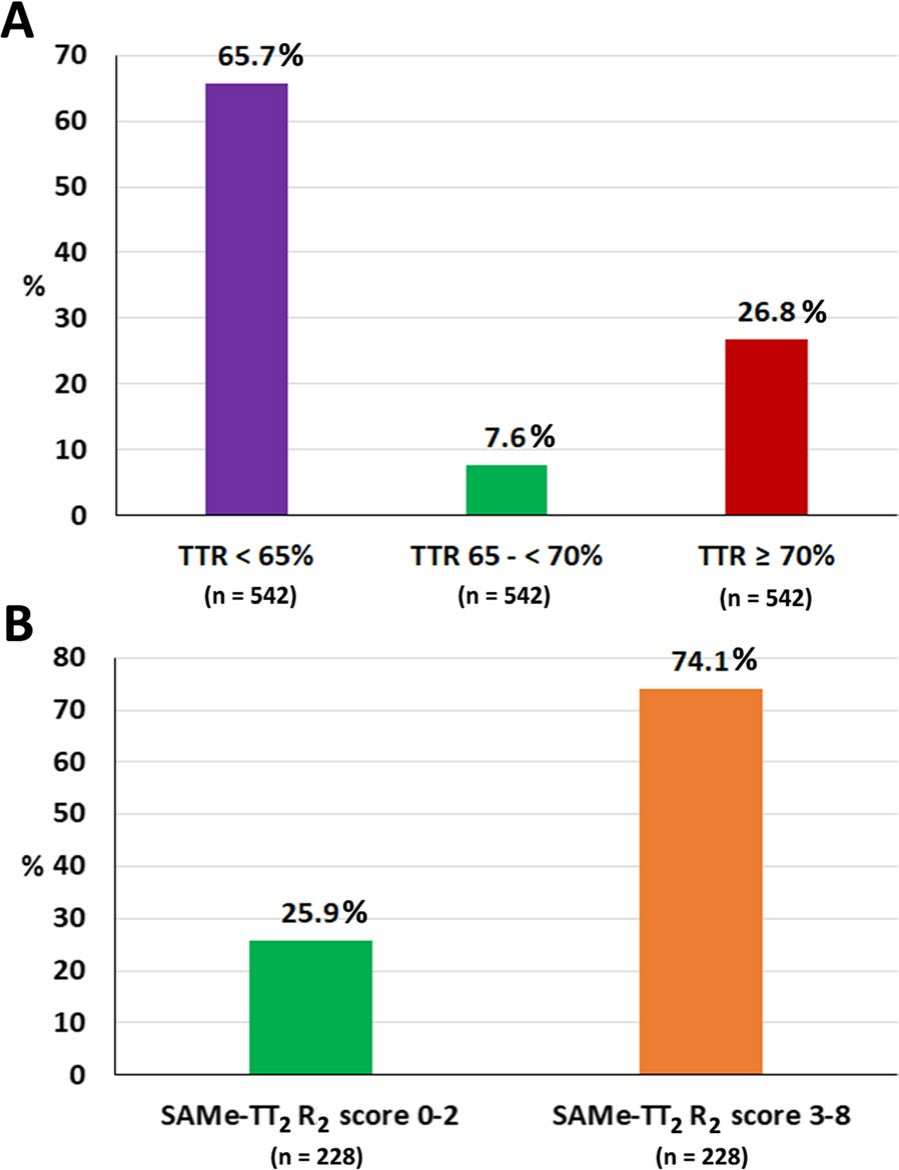
**A** Atrial fibrillation patients who were taking warfarin and who had a low SAMe-TT_2_R_2_ score (0–2) compared among different percentages of time in therapeutic range. **B** Atrial fibrillation patients who were taking NOACs compared between those with a SAMe-TT_2_R_2_ score 0–2 and those with a SAMe-TT_2_R_2_ score 3–8. Abbreviation: TTR, time in therapeutic range; NOACs, non-vitamin K antagonist oral anticoagulants; SAMe-TT_2_R_2_ score, S = female sex (1 point); A = age < 60 years (1 point); Me = medical history > 2 of the following: hypertension, diabetes, coronary artery disease/myocardial infarction, peripheral arterial disease, congestive heart failure, previous stroke, pulmonary disease, and hepatic or renal disease (1 point); T = treatment (interacting drugs, e.g., amiodarone for rhythm control) (1 point); T_2_ = tobacco use within 2 years (2 points); and, R_2_ = non-Caucasian race (2 points)

A total of 3461 AF patients were recruited into the COOL-AF Thailand registry during 2014–2020. Of those, 2568 patients who were taking OACs were eligible for inclusion in this study. There were 2340 patients taking warfarin, and 228 patients taking NOACs (83 for direct thrombin inhibitor, and 145 for Factor Xa inhibitors). Figure 1 shows a flow diagram of the study population and protocol. The average age of all patients was 68.8 ± 10.7 years. Most patients had hypertension (72.5%) or renal disease (54.2%). The average CHA_2_DS_2_-VASc, HAS-BLED, and SAMe-TT_2_R_2_ scores were 3.3 ± 1.6, 1.6 ± 1.0, and 3.1 ± 0.8, respectively. Only 12% of overall patients were also taking antiplatelet drugs. Most patients taking warfarin had a TTR < 65% (65.1%). Figure 2A shows AF patients who were taking warfarin and who had a low SAMe-TT_2_R_2_ score (0–2) compared among different TTRs. Fifty-nine patients who were taking NOACs (25.9%) had a low SAMe-TT_2_R_2_ score. Figure 2B shows the distribution of AF patients who were taking NOACs compared between those with a SAMe-TT_2_R_2_ score 0–2 and those with a SAMe-TT_2_R_2_ score 3–8. Patient baseline demographic and clinical data are shown in Table 1.

**Table 1:** Baseline characteristics of the study population

Characteristics	All (N = 2568)	Warfarin group (n = 2340)	NOACs group (n = 228)	*p* value
Age (years)	68.8 ± 10.7	68.8 ± 10.7	68.5 ± 10.6	0.701
Male sex	1453 (56.6%)	1323 (56.5%)	130 (57.0%)	0.889
Medical history				
Hypertension	1862 (72.5%)	1710 (73.1%)	152 (66.7%)	***0.039***
Diabetes	690 (26.9%)	637 (27.2%)	53 (23.2%)	0.196
CAD/previous MI	416 (16.2%)	378 (16.2%)	38 (16.7%)	0.841
Peripheral arterial disease	32 (1.2%)	31 (1.3%)	1 (0.4%)	0.357
Congestive heart failure	702 (27.3%)	660 (28.2%)	42 (18.4%)	***0.002***
Previous ischemic stroke/TIA	538 (21.0%)	502 (21.5%)	36 (15.8%)	***0.045***
Pulmonary disease	24 (0.9%)	24 (1.0%)	0 (0.0%)	0.265
Hepatic disease	24 (0.9%)	23 (1.0%)	1 (0.4%)	0.717
Renal disease	1392 (54.2%)	1287 (55.0%)	105 (46.1%)	***0.010***
CHA_2_DS_2_-VASc score	3.3 ± 1.6	3.3 ± 1.6	3.0 ± 1.6	***0.001***
HAS-BLED score	1.6 ± 1.0	1.6 ± 1.0	1.1 ± 0.8	** < *0.001***
SAMe-TT_2_R_2_ score	3.1 ± 0.8	3.1 ± 0.8	3.1 ± 0.9	0.685
SAMe-TT_2_R_2_ score 0–2	617 (24.0%)	558 (23.8%)	59 (25.9%)	0.493
Components of SAMe-TT_2_R_2_ score				
Female sex	1115 (43.4%)	1017 (43.5%)	98 (43.0%)	0.889
Age < 60 years	491 (19.1%)	443 (18.9%)	48 (21.1%)	0.437
Medical history > 2 comorbidities	957 (37.3%)	893 (38.2%)	64 (28.1%)	***0.003***
Interacting drug treatment	132 (5.1%)	107 (4.6%)	25 (11.0%)	** < *0.001***
Tobacco use within 2 years	59 (2.3%)	54 (2.3%)	5 (2.2%)	0.912
Non-Caucasian race	–	–	–	–
Serum creatinine (mg/dL)	1.3 ± 2.5	1.3 ± 2.7	1.1 ± 0.3	0.235
LVEF (%)	59.7 ± 14.0	59.5 ± 14.2	61.3 ± 12.6	***0.043***
TTR (%)	52.1 ± 27.4	52.1 ± 27.4	–	–
TTR < 65%	1494 (65.1%)	1494 (65.1%)	–	–
TTR 65 to < 70%	168 (7.3%)	168 (7.3%)	–	–
TTR ≥ 70%	633 (27.6%)	633 (27.6%)	–	–
Antithrombotic medications				
Antiplatelet	309 (12.0%)	294 (12.6%)	15 (6.6%)	***0.008***
Aspirin	264 (10.3%)	252 (10.8%)	12 (5.3%)	***0.009***
P2Y_12_ inhibitors	81 (3.2%)	77 (3.3%)	4 (1.8%)	0.205

*NOACs* non-vitamin K oral anticoagulants, *SD* standard deviation, *CAD* coronary artery disease, *MI* myocardial infarction, *TIA* transient ischemic attack, *LVEF* left ventricular ejection fraction, *TTR* time in therapeutic range)

A *p* value < 0.05 indicates statistical significance (bold and italic)

Variables are shown as mean ± SD or number (%)

### All OAC patients

**Fig. 3:**
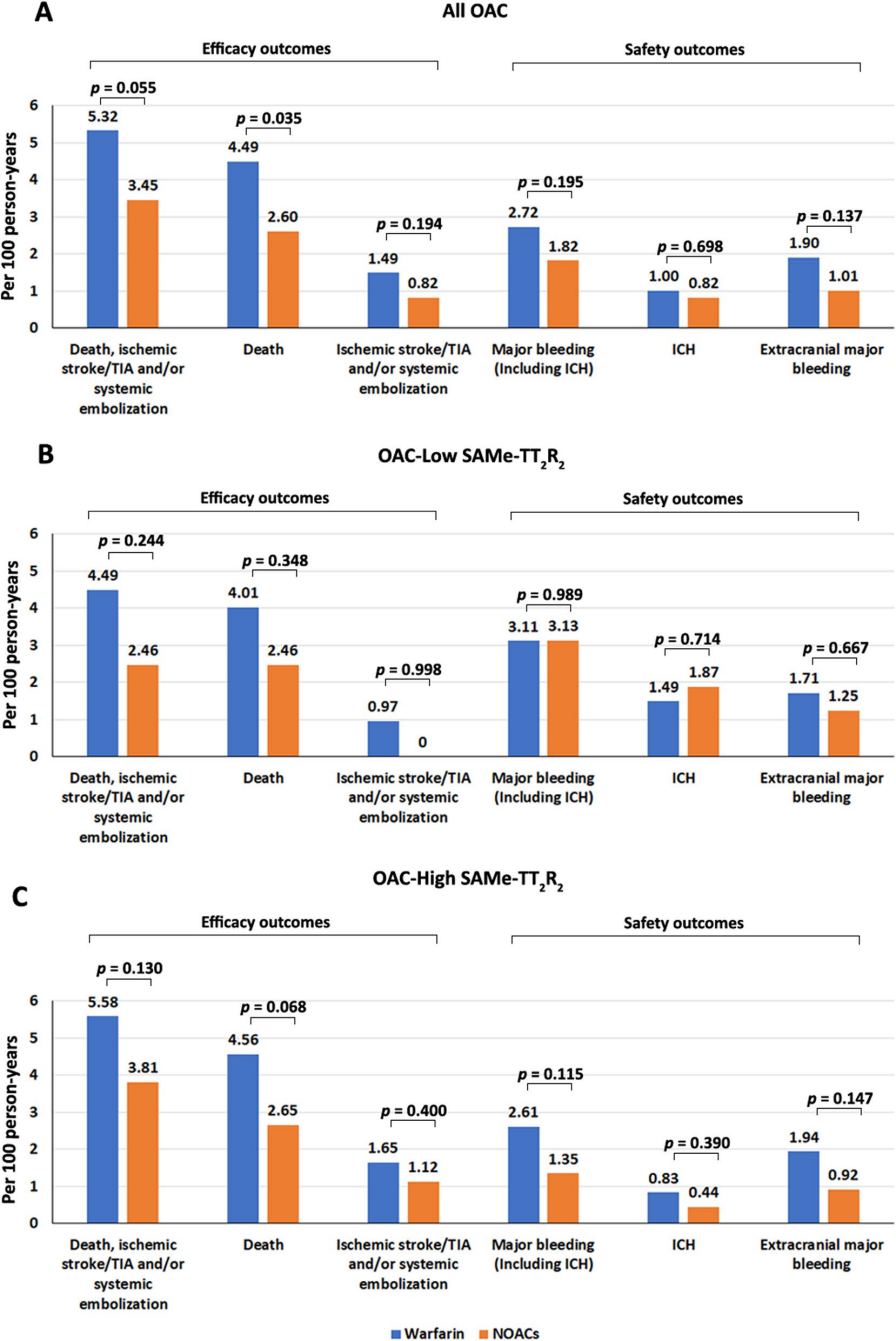
Cumulative incidence of efficacy and safety outcomes of atrial fibrillation patients compared between those taking warfarin and those taking NOACs among **A** all patients taking oral anticoagulants, **B** patients with a low SAMe-TT_2_R_2_ score (range: 0–2), and **C** patients with a high SAMe-TT_2_R_2_ score (range: 3–8)

Table 2 shows the incidence of primary and secondary outcomes compared between the warfarin and NOAC groups among all patients taking OAC, as well as those with low and high SAMe-TT_2_R_2_ score. Among overall patients and regardless of SAMe-TT_2_R_2_ score, 305 patients (13.0%) in the warfarin group and 21 patients (9.2%) in NOACs group had the primary efficacy outcome criteria. There was a trend towards increased primary efficacy outcome in warfarin group (hazard ratio [HR] 1.54, 95% confidence interval [CI] 0.99–2.40; *p* = 0.055), a significant increase in all-cause death (HR 1.71, 95%CI 1.03–2.83; *p* = 0.038), and no significant difference in IS/TIA and/or SE (HR 1.83; 95%CI 0.74–4.50; *p* = 0.190) compared to NOACs (Table 2, Fig. 3A). After adjustment for potential confounders, there was no significant difference in the primary efficacy outcome (adjusted [aHR] 1.43, 95%CI 0.90–2.26; *p* = 0.127) a trend towards increased all-cause death (aHR 1.55; 95%CI 0.93–2.57; *p* = 0.094) in patients taking warfarin compared to NOACs (Table 3).

**Table 2:** Incidence of primary and secondary outcomes compared between the warfarin with NOAC groups among all patients taking OAC, as well as patients with a low and high SAMe-TT2R2 score

Outcomes	All OAC (N = 2568)					
	Warfarin (n = 2340)		NOACs (n = 228)		HR (95%CI)	*p* value
	Number of events n (%)	Incidence per 100 person-years	Number of events n (%)	Incidence per 100 person-years		
Primary efficacy outcome^a^	305 (13.0%)	5.32	21 (9.2%)	3.45	1.54 (0.99–2.40)	0.055
All-cause death	257 (11.0%)	4.49	16 (7.0%)	2.60	1.71 (1.03–2.83)	***0.038***
IS/TIA and/or SE	86 (3.7%)	1.49	5 (2.2%)	0.82	1.83 (0.74–4.50)	0.190
Primary safety outcome^b^	155 (6.6%)	2.72	11 (4.8%)	1.82	1.50 (0.81–2.76)	0.198
Intracranial hemorrhage	57 (2.4%)	1.0	5 (2.2%)	0.82	1.19 (0.48–2.97)	0.708
Extracranial major bleeding	98 (4.2%)	1.9	6 (2.6%)	1.01	1.88 (0.82–4.28)	0.135
Secondary outcome						
CV death^c^	21 (0.9%)	0.36	0 (0.0%)	0	–	–
CV death^c^ or IS/TIA/SE	155 (6.6%)	2.69	10 (4.4%)	1.64	1.64 (0.87–3.11)	0.130
CV death3, IS/TIA/SE or major bleeding	267 (11.4%)	4.75	17 (7.5%)	2.83	1.68 (1.03–2.74)	***0.039***

Outcomes	OAC-Low SAMe-TT_2_R_2_ (n = 617)					
	Warfarin (n = 558)		NOACs (n = 59)		HR (95%CI)	*p* value
	Number of events n (%)	Incidence per 100 person-years	Number of events n (%)	Incidence per 100 person-years		
Primary efficacy outcome^a^	60 (10.8%)	4.49	4 (6.8%)	2.46	1.81 (0.66–4.97)	0.252
All-cause death	54 (9.7%)	4.01	4 (6.8%)	2.46	1.61 (0.58–4.45)	0.358
IS/TIA and/or SE	13 (2.3%)	0.97	0 (0.0%)	0	–	**–**
Primary safety outcome^b^	41 (7.3%)	3.11	5 (8.5%)	3.13	1.00 (0.40–2.53)	0.999
Intracranial hemorrhage	20 (3.6%)	1.49	3 (5.1%)	1.87	0.79 (0.23–2.65)	0.700
Extracranial major bleeding	21 (3.8%)	1.71	2 (3.4%)	1.25	1.43 (0.34–6.09)	0.630
Secondary outcome						
CV death^c^	5 (0.9%)	0.37	0 (0.0%)	0	–	–
CV death^c^ or IS/TIA/SE	29 (5.2%)	2.16	1 (1.7)	0.62	3.49 (0.48–25.64)	0.219
CV death^c^, IS/TIA/SE or major bleeding	58 (10.4%)	4.43	5 (8.5%)	3.13	1.42 (0.57–3.55)	0.448

Outcomes	OAC-High SAMe-TT_2_R_2_ (n = 1951)					
	Warfarin (n = 1782)		NOACs (n = 169)		HR (95%CI)	*p* value
	Number of events n (%)	Incidence per 100 person-years	Number of events n (%)	Incidence per 100 person-years		
Primary efficacy outcome^a^	245 (13.7%)	5.58	17 (10.1%)	3.81	1.47 (0.89–2.40)	0.128
All-cause death	203 (11.4%)	4.56	12 (7.1%)	2.65	1.73 (0.96–3.09)	0.066
IS/TIA and/or SE	73 (4.1%)	1.65	5 (3.0%)	1.12	1.48 (0.60–3.67)	0.396
Primary safety outcome^b^	114 (6.4%)	2.61	6 (3.6%)	1.35	1.93 (0.85–4.38)	0.117
Intracranial hemorrhage	37 (2.1%)	0.83	2 (1.2%)	0.44	1.86 (0.45–7.71)	0.394
Extracranial major bleeding	77 (4.3%)	1.94	4 (2.4%)	0.92	2.10 (0.77–5.74)	0.148
Secondary outcome						
CV death^c^	16 (0.9%)	0.36	0 (0.0%)	0	–	–
CV death^c^ or IS/TIA/SE	126 (7.1%)	2.85	9 (5.3%)	2.02	1.42 (0.72–2.78)	0.314
CV death^c^, IS/TIA/SE or major bleeding	209 (11.7%)	4.85	12 (7.1%)	2.72	1.78 (1.00–3.18)	0.052

*NOAC* non-vitamin K antagonist oral anticoagulant, *OAC* oral anticoagulant, *HR* hazard ratio, *CI* confidence interval, *IS* ischemic stroke, *TIA* transient ischemic attack, *SE* systemic embolism, *CV* cardiovascular, *SAMe-TT*_*2*_*R*_*2*_ score, *S* female sex (1 point), *A* age < 60 years (1 point), *Me* medical history > 2 of the following: hypertension, diabetes, coronary artery disease/myocardial infarction, peripheral arterial disease, congestive heart failure, previous stroke, pulmonary disease, and hepatic or renal disease (1 point), *T* treatment (interacting drugs, e.g., amiodarone for rhythm control) (1 point), *T*_*2*_ tobacco use within 2 years (2 points), *R*_*2*_ non-Caucasian race (2 points)

A *p* value < 0.05 indicates statistical significance (bold and italic)

^a^Primary efficacy outcome, including death, IS/TIA, and/or SE

^b^Primary safety outcome, including major bleeding

^c^CV death, including IS/TIA, myocardial infarction, and/or SE

**Table 3:** Primary and secondary outcomes of patients with atrial fibrillation after adjustment for potential confounders

Outcomes	All OAC (N = 2568)	
	Warfarin vs. NOACs	
	Adjusted hazard ratio (95%CI)	*p* value
Primary efficacy outcome^a^	1.43 (0.90–2.26)	0.127
All-cause death	1.55 (0.93–2.57)	0.094
IS/TIA and/or SE	1.89 (0.69–5.21)	0.217
Primary safety outcome^b^	1.55 (0.79–3.06)	0.204
Intracranial hemorrhage	1.33 (0.48–3.69)	0.587
Extracranial major bleeding	1.86 (0.75–4.61)	0.180
Secondary outcome		
CV death^c^	–	–
CV death^c^ or IS/TIA/SE	1.56 (0.79–3.07)	0.200
CV death^c^ or IS/TIA/SE or major bleeding	1.74 (1.01–2.99)	***0.045***

Outcomes	OAC-Low SAMe-TT_2_R_2_ (n = 617)	
	Warfarin vs. NOACs	
	Adjusted hazard ratio (95%CI)^d^	*p* value
Primary efficacy outcome^a^	1.60 (0.58–4.45)	0.367
All-cause death	1.49 (0.54–4.16)	0.444
IS/TIA and/or SE	–	**–**
Primary safety outcome^b^	1.41 (0.43–4.62)	0.574
Intracranial hemorrhage	0.92 (0.21–4.08)	0.910
Extracranial major bleeding	2.23 (0.29–16.89)	0.439
Secondary outcome		
CV death^c^	–	**–**
CV death^c^ or IS/TIA/SE	3.01 (0.40–22.33)	0.282
CV death^c^ or IS/TIA/SE or major bleeding	1.95 (0.61–6.31)	0.263

Outcomes	OAC-High SAMe-TT_2_R_2_ (n = 1951)	
	Warfarin vs. NOACs	
	Adjusted hazard ratio (95%CI)	*p* value
Primary efficacy outcome^a^	1.36 (0.81–2.26)	0.245
All-cause death	1.53 (0.85–2.76)	0.157
IS/TIA and/or SE	1.58 (0.57–4.38)	0.379
Primary safety outcome^b^	1.62 (0.71–3.70)	0.257
Intracranial hemorrhage	1.69 (0.40–7.09)	0.473
Extracranial major bleeding	1.69 (0.61–4.68)	0.310
Secondary outcome		
CV death^c^	–	–
CV death^c^ or IS/TIA/SE	1.38 (0.67–2.84)	0.387
CV death^c^ or IS/TIA/SE or major bleeding	1.67 (0.90–3.08)	0.103

*OAC* oral anticoagulant, *NOAC* non-vitamin K antagonist oral anticoagulant, *CI* confidence interval, *IS* ischemic stroke, *TIA* transient ischemic attack, *SE* systemic embolism, *CV* cardiovascular, *SAMe-TT*_*2*_*R*_*2*_ score, *S* female sex (1 point), *A* age < 60 years (1 point), *Me* medical history > 2 of the following: hypertension, diabetes, coronary artery disease/myocardial infarction, peripheral arterial disease, congestive heart failure, previous stroke, pulmonary disease, and hepatic or renal disease (1 point), *T* treatment (interacting drugs, e.g., amiodarone for rhythm control) (1 point), *T*_*2*_ tobacco use within 2 years (2 points), *R*_*2*_ non-Caucasian race (2 points)

A *p *value < 0.05 indicates statistical significance (bold and italic)

^a^Primary efficacy outcome, including death, IS/TIA, and/or SE

^b^Primary safety outcome, including major bleeding

^c^CV death, including IS/TIA, myocardial infarction, and/or SE

Among overall patients regardless of SAMe-TT_2_R_2_ score, 155 patients (6.6%) in the warfarin group and 11 patients (4.8%) in the NOACs group had the primary safety outcome criteria. There was no significant difference in the primary safety outcome between warfarin and NOACs both for unadjusted (HR 1.50, 95%CI 0.81–2.76; *p* = 0.198) and adjusted outcome (aHR 1.55, 95%CI 0.79–3.06; *p* = 0.204) analysis (Tables 2, 3).

### OAC patients with low SAMe-TT_2_R_2_ score

Among the patients with a low SAMe-TT_2_R_2_ score, 60 patients (10.8%) had the primary efficacy outcome criteria (4.49 per 100 person-years) in the warfarin group and 4 patients (6.8%) in the NOACs group (2.46 per 100 person-years) (Table 2, Fig. 3B). There was no significant difference in the primary efficacy outcome between warfarin group and NOAC group both unadjusted (HR 1.81, 95%CI 0.66–4.97; *p* = 0.252) and adjusted (aHR 1.60, 95%CI 0.58–4.45; *p* = 0.367) analysis (Tables 2, 3). Primary safety outcomes were reached in 41 patients (7.3%) in the warfarin group and 5 patients (8.5%) in NOACs group. There was no significant difference in the primary safety outcome for unadjusted (HR 1.00, 95%CI 0.40–2.53; *p* = 0.999), and adjusted (aHR 1.60, 95%CI 0.58–4.45; *p* = 0.367) analysis (Tables 2, 3).

### OAC patients with high SAMe-TT_2_R_2_ score

Among the patients with a high SAMe-TT_2_R_2_ score, the primary efficacy outcome criteria were reached in 245 patients (13.7%) in the warfarin group and 17 patients (10.1%) in NOAC group. There was no significant difference in the primary efficacy outcome (HR 1.47, 95%CI 0.89–2.40; *p* = 0.128), with a trend towards increased all-cause death in warfarin group both unadjusted (HR 1.73, 95%CI 0.96–3.09; *p* = 0.066), and adjusted (aHR 1.53, 95%CI 0.85–2.76; *p* = 0.157) analysis (Tables 2, 3 and Fig. 3C). The primary safety outcome criteria were reached in 114 patients (6.4%) in the warfarin group and 6 patients (3.6%) in the NOACs group. There was no significant difference in the primary safety outcome in warfarin and NOACs both unadjusted (HR 1.93, 95% CI 0.85–4.38; *p* = 0.117) and adjusted analysis (aHR 1.62, 95%CI 0.71–3.70; *p* = 0.257) (Tables 2, 3, Fig. 3C).

Our analysis of the secondary outcome after adjustment for potential confounders revealed that overall patients taking warfarin had significantly more CV death or IS/TIA/SE or major bleeding than those taking NOACs (11.4% vs. 7.5%, respectively; aHR 1.74, 95%CI 1.01–2.99; *p* = 0.045) regardless of SAMe-TT_2_R_2_ score. There was no significant difference in CV death or IS/TIA/SE or major bleeding between the two OAC groups when stratified as having a low or high SAMe-TT_2_R_2_ score (Table 3).

## Discussion

The results of this multicenter nationwide prospective study revealed no statistically significant difference in all-cause death, IS/TIA and/or SE, ICH or major bleeding compared between those taking warfarin and those taking NOACs after adjustment for potential confounders among overall patients, among patients with a low SAMe-TT_2_R_2_ score, and among patients with a high SAMe-TT_2_R_2_ score. However, the composite outcome of CV death or IS/TIA/SE or major bleeding significantly increased among overall patients that took warfarin compared to those that took NOACs. Our analysis stratified by low or high SAMe-TT2R2 score revealed no significant differences in outcomes between the warfarin and NOAC groups after adjustment for potential confounders.

Previous studies reported the SAMe-TT_2_R_2_ score to be related to adverse cardiovascular events, IS, major bleeding, and death [16, 18, 26]. Those studies reflected anticoagulant patients with AF having poor anticoagulation control appeared to be more thromboembolic, major bleeding and/or death in high score patients in addition to suboptimal TTR. Moreover, previous several studies reported that a SAMe-TT_2_R_2_ score of 2 or less could predict poor quality of anticoagulation control in AF patients taking warfarin [16–21]. To date, the SAMe-TT_2_R_2_ score has been recommended for predicting poor TTR in AF patients taking warfarin [1]. However, no study has investigated the efficacy and safety outcomes of AF patients compared between those taking warfarin and NOACs based on SAMe-TT_2_R_2_ score. It has been reported that NOACs were associated with a comparable rate of ischemic stroke, a reduced rate of ICH, and no significant increase in major bleeding when compared with warfarin [27–30]. However, none of those studies described whether the SAMe-TT_2_R_2_ score affected the outcome of the NOACs trial. The aim of the present study was to evaluate the predictive value of the SAMe-TT_2_R_2_ score in this clinical setting.

About a quarter of overall patients with warfarin and NOACs had low SAMe-TT_2_R_2_ score. However, most patients with warfarin had poor quality of anticoagulation control (TTR < 65%) despite having low SAMe-TT_2_R_2_ score. This reflected that SAMe-TT_2_R_2_ score was not a good predictive model for anticoagulation control leading to more CV death or IS/TIA/SE or major bleeding in warfarin patients. However, there was no significant difference in major bleeding (mostly extracranial major bleeding) among patients who were receiving warfarin and NOACs.

In patients with low SAMe-TT_2_R_2_ score, those taking warfarin had no significant difference in primary efficacy, primary safety and secondary outcomes. About 73% of patients prescribing warfarin reached TTR < 70% leading to increase efficacy and safety outcome in low SAMe-TT_2_R_2_ patients. This has been illustrated by previous studies that have illustrated that poor TTR is associated with thromboembolism, bleeding and/or mortality [31, 32]. In addition, the quality of anticoagulation control in warfarin patients can be evaluated by TTR, but is usually not routinely evaluated the anticoagulant level in NOACs patients in clinical practice. The appropriate NOACs level or optimal dose in each patient profile will be needed to evaluate in the future study. This led to non-significant difference in all outcomes between patients with warfarin and NOACs. Current European guidelines recommend the use of VKAs as a treatment alternative in patients with a low SAMe-TT_2_R_2_ score [1].

SAMe-TT_2_R_2_ score is not use to guide whether patients should be on OAC. It is used to predict the suboptimal INR control [1, 2, 33]. Therefore, if the chance of suboptimal INR is high, patients should not be on warfarin and NOACs were the preferred choice. In fact, NOACs is usually recommended as the preferred option but in some situation especially in country where cost of medication is concerned, consideration for warfarin use might be the issue. It should be noted that after the importance of SAMe-TT_2_R_2_ score has been eliminated the role of CHA_2_DS_2_-VASc score should be emphasized further. CHA_2_DS_2_-VASc score has been recommended to identify patients with non-valvular AF with very low risk of stroke and has no need for OAC [1–3, 33]. Recent data suggested that it can also be used to predict prosthetic valve thrombosis among patients with mechanical mitral valve [34]. The score of 2.5 had been associated with increased risk of prosthetic valve thrombosis [34].

When we compared the efficacy and safety outcomes of patients with a high SAMe-TT_2_R_2_ score between the warfarin and NOAC groups, the outcomes were comparable to those observed among patients with a low SAMe-TT_2_R_2_ score. This indicates that the SAMe-TT_2_R_2_ score should not be used for OAC selection decision-making.

### Limitations

The mentionable limitation in this study is there was a low event rate for CV death among patients taking warfarin, and no CV death in patients taking NOACs. As such, even though we enrolled a large study population, a much larger study population may be needed to more accurately examine CV death as an outcome variable. Another limitation is this study enrolled only Thai AF patients, so our results may not be generalizable to other races. The finding that there was no statistically significant difference in the efficacy and safety outcomes between patients who took warfarin and patients who took NOACs has to be interpreted with caution. This study had a small sample size of patients in the NOAC group and in patients with low SAMe-TT_2_R_2_ score groups. Therefore, it may not be enough to demonstrate the significant difference between the comparison group.

## Conclusions

AF patients taking warfarin had a significantly higher rate of CV death or IS/TIA/SE or major bleeding compared to those taking NOACs regardless of SAMe-TT_2_R_2_ score. The results of this study do not support the use of SAMe-TT_2_R_2_ score to guide OAC selection.

## Data Availability

The dataset that was used to support the results and conclusion of this study are included within the manuscript. Additional data are available upon contacting Rungroj Krittayaphong at rungroj.kri@mahidol.ac.th with the reasonable request.
